# A rare case of pituitrin-induced delayed encephalopathy

**DOI:** 10.12669/pjms.305.5491

**Published:** 2014

**Authors:** Tingting Xu, Lijun Zhu, Jiachun Feng, Shaokuan Feng

**Affiliations:** 1 Tingting Xu, MD PhD, Department of Neurology, The First Teaching Hospital of Jilin University, Changchun, China.; 2 Lijun Zhu, MD, PhD, Department of Neurology, The Third Teaching Hospital of Jilin University, Changchun, China.; 3 Jiachun Feng, MD, PhD, Department of Neurology, The First Teaching Hospital of Jilin University, Changchun, China.; 4 Shaokuan Fang, MD, PhD, Department of Neurology, The First Teaching Hospital of Jilin University, Changchun, China.

**Keywords:** Delayed encephalopathy, Hyponatremia, Pituitrin

## Abstract

A 26-year-old girl was admitted to the Neurological Department of The First Teaching Hospital of Jilin University with complaints of rapidly deteriorating speech clumsiness for 18 days. Five Days before her attack she had undergone intramuscular pituitrin therapy on account of recurrent haemoptysis. Cranial MRI revealed multiple abnormal signals in bilateral hemisphere and symmetric abnormal signal in bilateral caudate nucleus and putamen. Serum electrolyte analysis revealed mild hyponatremia. The abnormal signals in bilateral hemisphere almost disappeared after 7 days of cerebral circulation ameliorating and serum electrolyte turbulence correcting therapy, whereas the symmetric abnormal signals in bilateral caudate nucleus and putamen still existed. A diagnosis of delayed encephalopathy was made and we presume the encephalopathy was associated with pituitrin therapy.

## INTRODUCTION

Pituitrin which has been proven effective for the treatment of hemorrhagic disease contains antidiuretic hormone and oxytocin. Antidiuretic hormone also called vasopressin has been shown to induce vasoconstriction and might cause a slight increase in blood pressure.^1^

Although rare, vasopressin has also been shown to cause hyponatremia^1^^,^^2^ which may lead to serious complications, such as hyponatremia encephalopathy.^3^ We herein report the case of a patient who suffered from late onset encephalopathy caused by vasoconstriction as well as hyponatremia following the use of pituitrin and also investigate the possible pathogenesis.

## CASE REPORT

**Fig.1:**
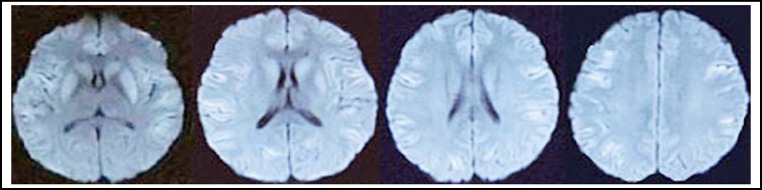
DWI (2013-05-31) showed multiple abnormal signals in bilateral hemisphere and symmetric abnormal signal in bilateral caudate nucleus and putamen

**Fig.2:**
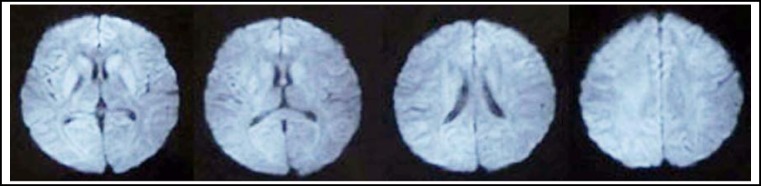
DWI (2013-06-07) showed symmetric abnormal signals in bilateral caudate nucleus and putamen

A 26-year-old girl was admitted to the Neurological Department of the First Teaching Hospital of Jilin University with complaints of rapidly deteriorating speech clumsiness for 18 days. Five Days before her attack she had undergone intramuscular pituitrin therapy (12U twice a day for 4 days and 12U once a day for 1 day) on account of recurrent haemoptysis. Prior to this, she had been healthy. Cranial MRI (2013-05-31 Fig.1) revealed multiple abnormal signals in bilateral hemisphere and symmetric abnormal signal in bilateral caudate nucleus and putamen. In our hospital, blood studies including complete blood count, liver, renal, thyroid and parathyroid function tests, blood glucose and autoimmune tests were all normal. Serum electrolyte analysis revealed mild hyponatremia (129 mEq/L). Cerebral spinal fluid tests and Electroencephalogram were also normal. The abnormal signals in bilateral hemisphere almost disappeared after 7 days of cerebral circulation ameliorating and serum electrolyte turbulence correcting therapy, whereas the symmetric abnormal signals in bilateral caudate nucleus and putamen still existed. (2013-06-07 Fig.2) Based on the aforementioned radiologic and clinical features, a diagnosis of delayed encephalopathy was made and we presume the encephalopathy was associated with pituitrin therapy.

## DISCUSSION

Pituitrin contains vasopressin and oxytocin, which have very similar structures. The name ‘vasopressin’ made it possible to refer to a hormone that is capable of both increasing arterial pressure and triggering capillary vasoconstriction in humans.^4^ Such effects are only observed at high doses. High doses of pituitrin therapy for haemoptysis may result in cerebral arteriolar and capillary contraction. Vasopressin levels of more than 20-30 pg/ml produce a pressor response with causing organ hypoperfusion.^5^ Due to a wide range of collateral circulation in cortex areas, blood supply in the cortex areas recovered soon. Furthermore cortex areas are more resistant to ischemia and therefore no permanent infarct was bequeathed in these areas, whereas caudate nucleus and putamen lack collateral circulation and these areas are more sensitive to ischemia and therefore there remains infarct in these areas.

Vasopressin also inhibits urine output, earning it the name ‘antidiuretic hormone’. Excessive circulating levels of vasopressin results in hyponatremia by decreasing renal free water excretion. Acute hyponatremia(defined as hyponatremia developing within 48 h) especially with serum sodium level <125 mmol/l predominantly causes neurologic symptoms, including confusion, reversible ataxia, psychosis, lethargy and disorientation.^6^^,^^7^ In this case, hyponatremia was noticed after admission and we inferred that hyponatremia aggravated this patient’ s damage in the nervous system.

In the light of this case, we emphasize that neurosurgeons and neurointensivists must be vigilant for the possibility of vasoconstriction and hyponatremia during pituitrin treatment.
